# Studying the Effect of MBNL1 and MBNL2 Loss in Skeletal Muscle Regeneration

**DOI:** 10.3390/ijms25052687

**Published:** 2024-02-26

**Authors:** Ramesh S. Yadava, Mahua Mandal, Mani S. Mahadevan

**Affiliations:** Department of Pathology, University of Virginia, Charlottesville, VA 22908, USA; ry3b@virginia.edu (R.S.Y.);

**Keywords:** muscle regeneration, myotonic dystrophy, RNA toxicity, satellite cells, muscleblind, RNA binding proteins, muscular dystrophy

## Abstract

Loss of function of members of the muscleblind-like (MBNL) family of RNA binding proteins has been shown to play a key role in the spliceopathy of RNA toxicity in myotonic dystrophy type 1 (DM1), the most common muscular dystrophy affecting adults and children. MBNL1 and MBNL2 are the most abundantly expressed members in skeletal muscle. A key aspect of DM1 is poor muscle regeneration and repair, leading to dystrophy. We used a BaCl_2_-induced damage model of muscle injury to study regeneration and effects on skeletal muscle satellite cells (MuSCs) in *Mbnl1^∆E3^*^/*∆E3*^ and *Mbnl2^∆E2^*^/*∆E2*^ knockout mice. Similar experiments have previously shown deleterious effects on these parameters in mouse models of RNA toxicity. Muscle regeneration in *Mbnl1* and *Mbnl2* knockout mice progressed normally with no obvious deleterious effects on MuSC numbers or increased expression of markers of fibrosis. Skeletal muscles in *Mbnl1^∆E3^*^/*∆E3*^*/ Mbnl2^∆E2^*^/+^ mice showed increased histopathology but no deleterious reductions in MuSC numbers and only a slight increase in collagen deposition. These results suggest that factors beyond the loss of MBNL1/MBNL2 and the associated spliceopathy are likely to play a key role in the defects in skeletal muscle regeneration and deleterious effects on MuSCs that are seen in mouse models of RNA toxicity due to expanded CUG repeats.

## 1. Introduction

Myotonic dystrophy type 1 (DM1), the most common muscular dystrophy in adults and children, is caused by a (CTG)_n_ expansion within the 3′ untranslated region (3′UTR) of the dystrophia myotonica protein kinase (DMPK) gene [1,2]. DM1 is a multisystemic disorder affecting many tissues including skeletal muscle and cardiac tissues. The mutation results in nuclear retention of the mutant RNA forming RNA foci [3,4]. DM1 is the prototype for RNA toxicity as a pathogenic mechanism whereby the toxic RNA acts in a trans-dominant manner to affect cellular function. This first clear demonstration of RNA toxicity due to the mutant RNA was reported by the Mahadevan lab in 1998 [5]. Using C2C12 mouse myoblast cell lines, and Cre-Lox technology, they demonstrated deleterious effects of the mutant DMPK 3′UTR mRNA on myogenic differentiation [6]. Subsequently, the Mahadevan lab provided the first in vivo demonstration that RNA toxicity resulting in DM1 could potentially be reversed by silencing expression of the toxic RNA using a mouse model utilizing a tetracycline (Tet) inducible transgene [7]. This was followed by the first demonstration that antisense oligonucleotide (ASO) therapies targeting an RNA with an expanded (CUG) tract, in another mouse model of RNA toxicity in DM1 (the HSALR model [8]), could correct DM1 phenotypes [9].

The prevailing hypothesis for the mechanism of RNA toxicity in DM1 involves adverse effects on RNA binding proteins, primarily members of the muscleblind-like family of proteins (MBNL1-3) and CUG binding protein-1 (also known as CELF1) [10]. In addition to their roles as splicing factors, these proteins have a myriad of functions in RNA processing and localization [11]. There is also experimental evidence for other mechanisms involving transcription factors, signaling such as the glycogen synthase kinase 3-beta (GSK-3β), AKT, AMPK, PKC, Tweak/Fn14, PI3K/AKT pathways, other RNA binding proteins (e.g., hnRNPA1 and MSI2), circular RNA generation, microRNAs, mitochondrial dysfunction, increased cellular senescence, and most recently, calcium channel dysfunction in conjunction with chloride channel dysfunction [12,13,14,15,16,17,18,19,20,21,22,23,24,25,26,27,28,29,30,31,32,33,34]. However, the strongest evidence is for the loss of function of MBNL proteins due to binding to the mutant RNA and their sequestration in the RNA foci [35]. The hypothesis is strongly supported by mouse models which lack MBNL1 [36] and tissue-specific compound loss of MBNL1 and MBNL2 [37,38].

Skeletal muscle disease in DM1 is characterized by progressive wasting and dystrophy. Typically, in most muscular dystrophies, there is evidence of repeated rounds of degeneration and regeneration. In contrast, muscle histopathology in DM1 often shows a surprising lack of regenerative response despite ongoing dystrophy [39]. This is thought to reflect defects in myogenesis, growth, and regeneration of skeletal muscle as extensively reviewed by Andre et.al. and others [40,41,42]. The key to a proper regenerative response is muscle stem cells (MuSCs) also known as satellite cells that proliferate in response to damage and differentiate into myoblasts that become the source of new cells for muscle repair [43]. We recently modeled muscle regeneration in our DM200 mouse model of RNA toxicity [44]. The DM200 model expresses a tetracycline-inducible GFP transgene with a 3′ UTR consisting of the DMPK 3′UTR with (CTG)_200_ [7,45]. In that study, we found that the toxic RNA had deleterious effects on MuSCs [44]. The number of MuSCs was reduced by RNA toxicity as was their response to damage. This was associated with a delay in muscle repair and maturation in response to damage and a dystrophic response characterized by fat deposition and fibrosis. Of note, we proved that these effects were due to the toxic RNA by treating the mice with an antisense oligonucleotide (ASO) that targeted the DMPK 3′UTR and degraded the toxic RNA [44]. This resulted in a recovery of the MuSC numbers and their response to damage and correlated with reduced fibrosis and dystrophy.

MBNL1 and MBNL2 are the primary MBNL proteins in skeletal muscle, with MBNL1 being the predominant MBNL protein [37]. We previously reported that both proteins are also expressed in MuSCs in adult skeletal muscles [44]. In contrast, MBNL3 is expressed in multiple tissues during embryonic development but is not detected in adult skeletal muscle [46]. However, the authors found that MBNL3 deficiency was associated with impaired muscle regeneration in older (>8 months old) mice. A subsequent study showed that MBNL3 was not expressed in quiescent MuSCs but did become expressed in MuSCs that had begun differentiation to myoblasts [38]. Interestingly, MBNL3 had a narrow window of expression at 3 days post-injury that corresponds to a period in muscle regeneration when quiescent MuSCs are activated to differentiate to myoblasts [46].

The goal of the current study was to evaluate muscle regeneration in mouse models of MBNL1 and MBNL2 deficiency in order to assess if loss of MBNL proteins in skeletal muscle could account for the regeneration and satellite cell defects observed with RNA toxicity. Mice with deletion of exon 3 of *Mbnl1* (*Mbnl1^∆E3^*^/*∆E3*^) and deletion of exon 2 of *Mbnl2* (*Mbnl2^∆E2^*^/*∆E2*^) resulting in MBNL1 or MBNL2 deficiency, have been generated and characterized by the Swanson lab at the University of Florida in the past two decades [36,37,47]. These were generously provided to us for this study. Muscle regeneration has not been studied systematically in mice deficient for MBNL1 or MBNL2. In order to study the response to damage, we utilized a BaCl_2_-induced injury model of the tibialis anterior (TA) muscle, an experimental approach that has been standardized and used extensively over the last twenty years [48]. This is analogous to the approach used in our recent study on the DM200 model [44].

## 2. Results

### 2.1. MBNLs, Muscle Histology, and Fiber Size Analysis

**Figure 1:**
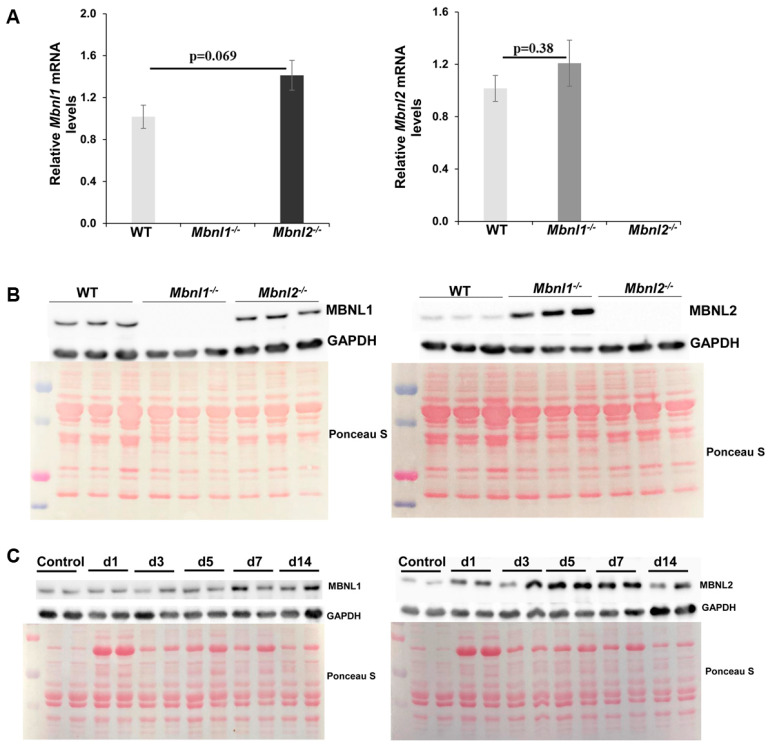
Characterization of *Mbnl*^−/−^ and *Mbnl2*^−/−^ mice. (**A**) Quantitative RT-PCR showed a small increase in the expression of *Mbnl1* mRNA in the TA muscle of *Mbnl2*^−/−^ knockout mice (*p* = 0.069) but no significant change in the expression of *Mbnl2* mRNA in the TA muscle of *Mbnl1*^−/−^ knockout mice (*p* = 0.38). (**B**) Immunoblot analysis of MBNL1 and MBNL2 in skeletal muscle (TA) from WT, *Mbnl1*^−/−^*,* and *Mbnl2*^−/−^ knockout 2-month-old mice. GAPDH was used as loading control; Ponceau S staining of blots; genotype of mice as indicated. (**C**) Immunoblot analysis showing expression levels of MBNL1 (left) and MBNL2 (right) in TA muscles of wildtype mice during muscle regeneration. GAPDH and Ponceau S are used as described above.

The mice used for all the studies were two months old, similar to our studies in the DM200 model. As a first step, we performed RT-PCR using primers in exon 2 and 3 of each gene, to confirm the deletion of the respective exons (*Mbnl1* exon 3 and *Mbnl2* exon 2) in the TA muscles of the knockout mice (Figure 1). As expected, RT-PCR demonstrated the absence of *Mbnl1^E3^* expression in MBNL1 knockout mice and no *Mbnl2^E2^* expression in MBNL2-deficient mice. Furthermore, it showed no significant change in expression of *Mbnl1* in MBNL2 knockout mice or *Mbnl2* in MBNL1 knockout mice (Figure 1A). Western blots showed an absence of MBNL2 in the MBNL2 knockout mice with no evident change in MBNL1 (Figure 1B). However, in the MBNL1 knockout mice, there was a two-to-three-fold increase in MBNL2 protein levels (Figure 1B), similar to previous results [37].

We then examined the levels of MBNL1 and MBNL2 at various time points after BaCl_2_-induced damage in the tibialis anterior (TA) muscle of wildtype mice (Figure 1C). BaCl_2_-induced damage is a commonly used approach to model skeletal muscle regeneration [48]. We found that MBNL1 levels began increasing at about 7 days post-damage, a period when regenerated fibers start to mature, with the elevated levels persisting to day 14 after damage, when maturation continues. MBNL2 levels increased at an earlier time point (3 days post-damage), a time when new muscle fibers are being generated and muscle fibers express more embryonic myosin isoforms. MBNL2 levels started to recede by day 14 post-damage, during the maturation phase.

**Figure 2:**
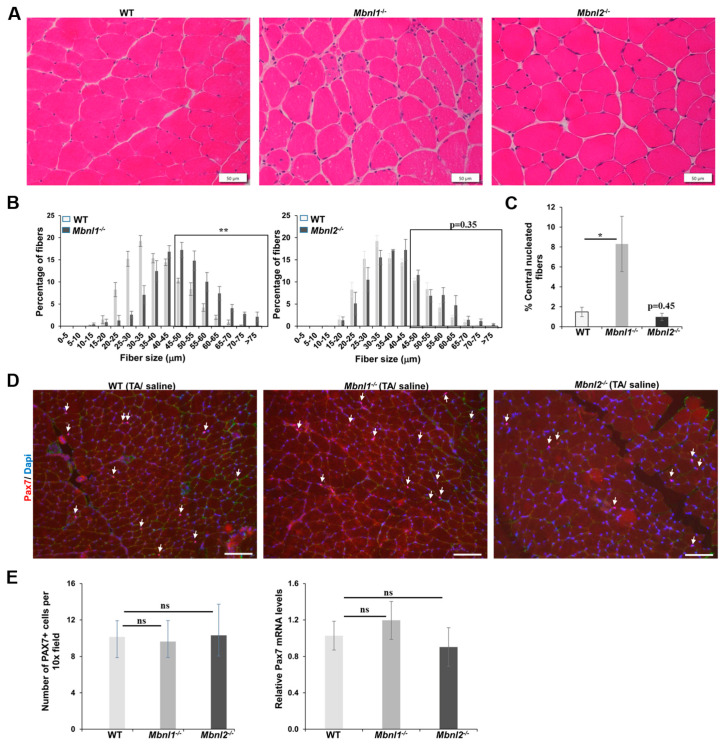
(**A**) Representative H&E staining of TA muscle sections of non-injured muscle and their fiber size distribution analysis in the WT, *Mbnl1*^−/−^*,* and *Mbnl2*^−/−^ mice; scale bar is 50 µm. (**B**) Fiber quantification showed an increase in the percentage of fibers >45 µm (boxed area) in the non-injured muscle of *Mbnl1*^−/−^ mice (*p* = 0.002) and no difference in *Mbnl2*^−/−^ non-injured muscle (*p* = 0.35) as compared to wildtype mice. *n* = 5 mice/group; ** *p* < 0.01; Student’s *t*-test; errors bars are mean ± SEM. (**C**) Quantification of centrally nucleated fibers in uninjured TA sections of WT, *Mbnl1*^−/−^, and *Mbnl2*^−/−^ mice. *n* ≥ 4 mice/group; * *p* < 0.05. (**D**) Immunofluorescence for PAX7 (red) in uninjured TA sections from wildtype, *Mbnl1*^−/−^*,* and *Mbnl2*^−/−^ mice; scale bar is 100 µm. Nuclei were stained with DAPI (blue). Arrows represent PAX7+ve cells. (**E**) (**Left**) Quantification showed no change in PAX7+ cells per 10x field in skeletal muscle (TA) of *Mbnl1*^−/−^ and *Mbnl2*^−/−^ as compared to wildtype mice. *n* = 3–4 mice/group; errors bars are mean ± SEM. (**Right**) qRT-PCR showed no change in the expression of Pax7 mRNA in skeletal muscle (TA) of *Mbnl1*^−/−^ and *Mbnl2*^−/−^ as compared to wildtype mice. *n* = 5 mice/group; ns = not significant; error bars are mean ± SEM. Abbreviations, TA—tibialis anterior; ns—not significant; WT—wildtype.

Next, we evaluated the histologic appearance of undamaged TA muscles from *Mbnl1* and *Mbnl2* knockout mice and compared them to wildtype mice (C57BL/6J) (Figure 2). H&E staining (Figure 2A) and subsequent fiber sizing of TA muscles from *Mbnl1*^−/−^ mice showed a significant (*p* = 0.002) increase in the percentage of larger fibers (>45 µm diameter) (Figure 2B). In contrast, there was no difference in fiber size in the TA muscles from *Mbnl2*^−/−^ mice as compared to wildtype mice (Figure 2B). Also, MBNL1 deficient mice showed clear evidence of pathology with an increased percentage of fibers with central nuclei (>8% of fibers in *Mbnl1*^−/−^ mice as compared to <2% in wildtype or *Mbnl2*^−/−^ mice; *p* < 0.05) (Figure 2C). We then evaluated if MBNL deficiency affected MuSCs or *Pax7* expression. Using immunofluorescence (IF) for PAX7 as a marker for MuSCs, we found no change in the number of MuSCs in *Mbnl1*^−/−^ or *Mbnl2*^−/−^ TA muscles as compared to wildtype mice (Figure 2D,E). Similarly, using qRT-PCR, we found no change in *Pax7* mRNA levels (Figure 2E).

### 2.2. MBNLs and Skeletal Muscle Regeneration

We then evaluated skeletal muscle generation in response to BaCl_2_-induced muscle damage in the *Mbnl1*^−/−^ and the *Mbnl2*^−/−^ mice and compared them to wildtype control mice. Such approaches have been well characterized and standardized to study the response to damage and subsequent repair and the associated histologic, molecular, and cellular responses [49,50,51,52,53]. BaCl_2_ causes rapid myonecrosis followed by an inflammatory response involving neutrophils and macrophages over the next 1–3 days at which point there is a dramatic increase in the number and activation of MuSCs. This is soon followed by a commitment of the satellite cells to a differentiated state. The satellite cells differentiate to myoblasts, which then differentiate and fuse, leading to the appearance of newly regenerating immature myofibers (marked by the expression of embryonic myosin heavy chain (MYH3)).

**Figure 3:**
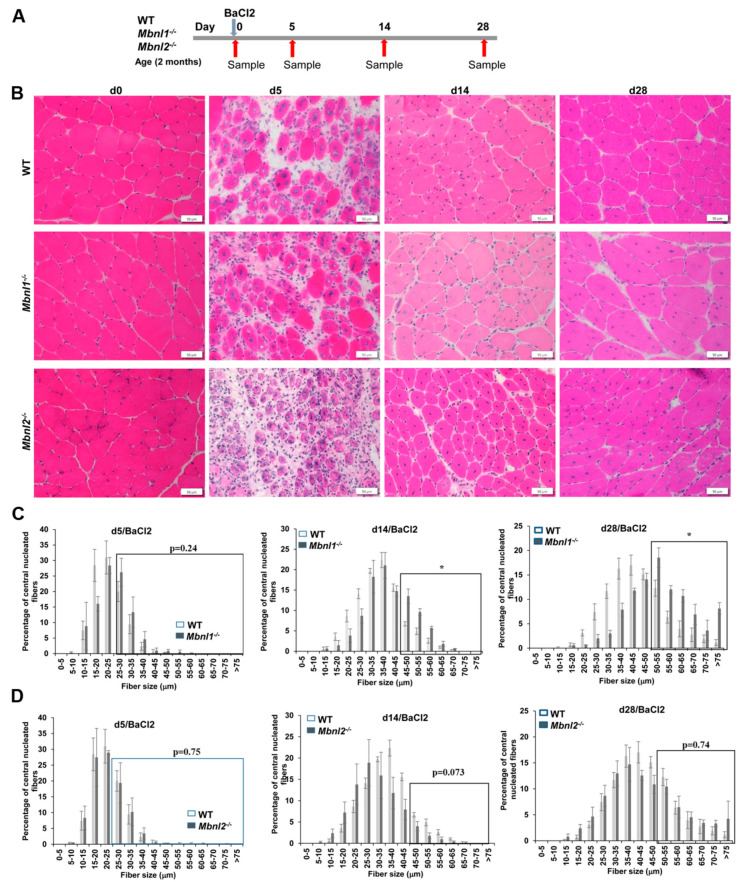
Muscle histology and fiber sizing confirms no change in muscle regeneration. (**A**) Schematic showing the experimental design used in this study. (**B**) Representative H&E staining of TA muscles sections 0, 5, 14, and 28 days post-injury (dpi) and their fiber size distribution analysis in the WT, Mbnl1^−/−^, and *Mbnl2*^−/−^ mice. *n* = 3–5 mice/group; scale bar is 50 µm. (**C**) Fiber size distribution analysis at 5 (fibers > 25 µm), 14(fibers > 45 µm), and 28 (fibers > 50 µm) days post-damage between WT and *Mbnl1*^−/−^ mice. *n* = 3–5 mice/group; * *p* < 0.05; Student’s *t*-test; *p*-values are indicated on the graphs; errors bars are mean ± SEM. (**D**) Fiber size distribution analysis at 5 (fibers > 25 µm), 14 (fibers > 45 µm), and 28 (fibers > 50 µm) days post-damage between WT and *Mbnl2*^−/−^ mice. *n* = 3–5 mice/group; * *p* < 0.05; Student’s *t*-test; *p*-values are indicated on the graphs; error bars are mean ± SEM.

Using male and female mice that were 2 months of age, we assessed the histology of the TA muscles at 0, 5, 14, and 28 days post-BaCl_2_ injury (Figure 3A) in areas where the damage was evident (i.e., myonecrosis and inflammation). At 5 days post-injury (dpi), H&E staining consistently showed evidence of regeneration with the presence of many small regenerating fibers with central nuclei (a marker of myofiber regeneration after damage) in all three groups (Figure 3B). By 14 days, fiber maturation and restoration of muscle morphology were well underway in all three groups, and by 28 days post-injury, things were almost back to pre-damage morphology (Figure 3B). There was an increase in fibers with central nuclei in all the groups, but that is a normal phenomenon of damage repair in mouse skeletal muscles. Of note, fiber sizing showed that damaged TA muscles in *Mbnl1*^−/−^ mice at 14 and 28 days post-injury showed a significantly increased proportion of fibers greater than 45–50 microns as compared to wildtype mice, similar to that observed in undamaged muscle from *Mbnl1*^−/−^ mice (Figure 3C). In the *Mbnl2*^−/−^ mice, there was no difference in fiber size distribution as compared to wildtype mice (Figure 3C).

**Figure 4:**
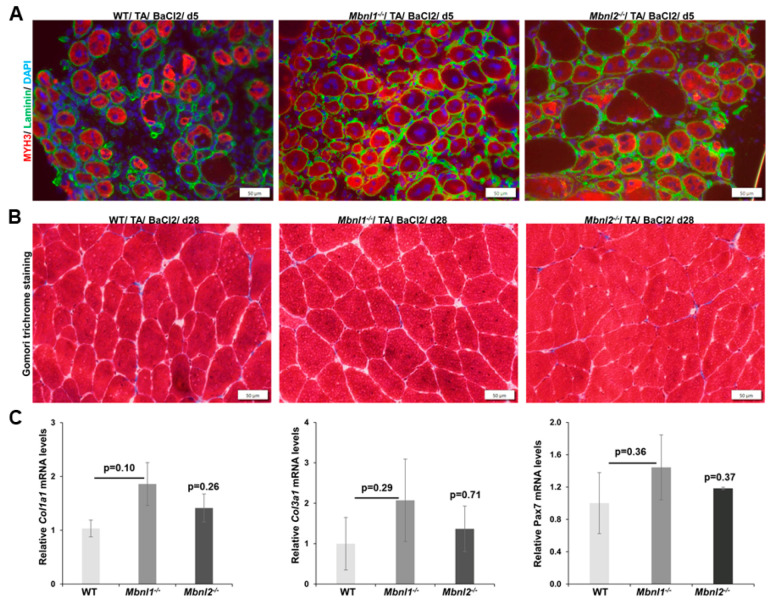
Muscle regeneration is not affected by the deletion of *Mbnl1*^−/−^ or *Mbnl2*^−/−^. (**A**) Immunofluorescence of MYH3 (red, regeneration marker) at 5 days post-damage showed maturing fibers in skeletal muscle (TA) of WT, *Mbnl1*^−/−^*,* and *Mbnl2*^−/−^ mice. Nuclei were stained with DAPI (blue), and laminin IF was used for outlining muscle fibers (green). Scale bar is 50 µm. (**B**) Gomori trichrome staining of representative TA muscle sections collected at 28 days post-damage showed similar collagen staining (blue stain) in the *Mbnl1*^−/−^ and *Mbnl2*^−/−^ mice as compared to wildtype (WT) mice. Scale bar is 50 µm. (**C**) Quantitative RT-PCR also showed no significant change in the expression of *Col1a1*, *Col3a1*, and *Pax7* mRNA in skeletal muscle (TA) of *Mbnl1*^−/−^ and *Mbnl2*^−/−^ mice as compared to wildtype mice at 28 days post-injury; p values for Student’s *t*-test as indicated relative to WT mice; *n* = 5 mice/group; error bars are mean ± SEM.

Next, we used immunofluorescence microscopy to determine the expression of embryonic myosin heavy chain (MYH3), a marker of nascent myofiber formation. We did this instead of RT-PCR, as the damage is localized and the MYH3 response is specifically relevant in the damaged areas. We did not observe any major differences in the MYH3 response with regard to the extent of MYH3 expression when comparing damaged TA muscles from wildtype mice and those from *Mbnl1*^−/−^ or *Mbnl2*^−/−^ mice (Figure 4A). A pathologic consequence of muscle damage and repair in muscular dystrophy is dystrophic change characterized by increased expression and deposition of collagen within the interstitial space. To assess this, we used Gomori trichrome staining (Figure 4B) to detect collagen in TA muscle sections collected 28 days post-injury in conjunction with RT-PCR for the expression of two collagen genes, *Co1la1* and *Col3a1* (Figure 4C). We found no evidence of increased collagen deposition or expression of the collagen genes in the *Mbnl1*^−/−^ or the *Mbnl2*^−/−^ mice as compared to wildtype mice. We also assessed *Pax7* expression at day 28 post-injury as mouse models of RNA toxicity including the HSALR and the DM200 mouse model have shown decreased levels of *Pax7* expression [30,44]. However, we did not find any evidence for decreased *Pax7* expression in either the *Mbnl1*^−/−^ or the *Mbnl2*^−/−^ models (Figure 4C).

### 2.3. Combined MBNL1 Loss with Hemizygous Loss of MBNL2

**Figure 5:**
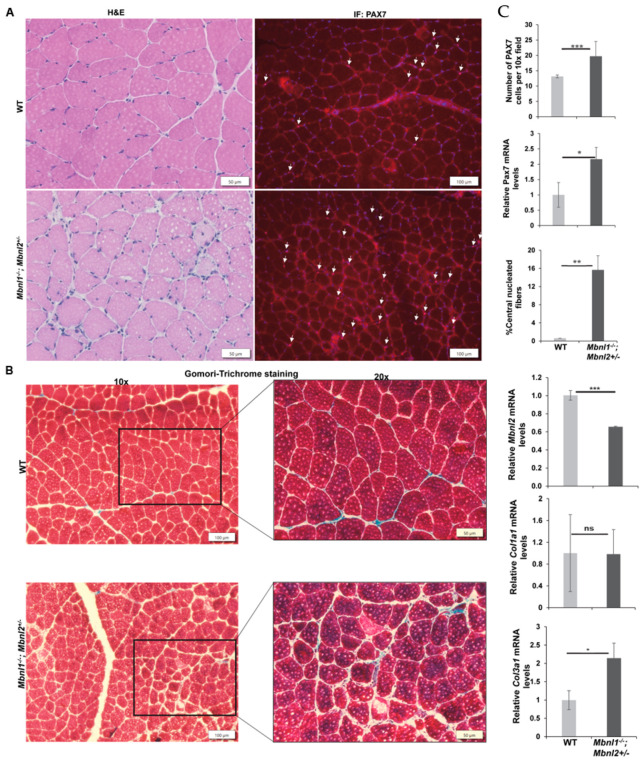
Combined deletion of *Mbnl1*^−/−^ and *Mbnl2*^+/−^. (**A**) H&E staining of WT and *Mbnl1*^−/−^*/Mbnl2*^+/−^ TA muscles showed centralized nuclei and small degenerating fibers in 2-month-old *Mbnl1*^−/−^*/Mbnl2*^+/−^ knockout mice. PAX7 IF (red) showing PAX7 + ve cells (arrows) in WT and *Mbnl1*^−/−^*/Mbnl2*^+/−^ TA muscles at 2 months of age; nuclei (blue). (**B**) Gomori trichrome staining of TA muscles showed no obvious increase in collagen staining (10×); higher magnification (20×) identified patches of increased staining in the *Mbnl1*^−/−^*/Mbnl2*^+/−^ TA muscles. (**C**) Increased PAX7 + cells, Pax7 mRNA, and **c**entral nucleated fibers in TA muscles of *Mbnl1*^−/−^*/Mbnl2*^+/−^ mice (*n* = 5) as compared to WT (*n* = 4). Quantitative RT-PCR showed a significant decrease in *Mbnl2* mRNA levels (*p* = 0.00018), no change in the expression of *Col1a1*, and a significant increase in the expression of *Col3a1* (*p* = 0.039); error bars are mean ± SEM. Scale bars indicated. * *p* < 0.05, ** *p* < 0.01, *** *p* < 0.001, ns = not significant for Student’s *t*-test.

MBNL2 is known to be upregulated in MBNL1-deficient mice and compensates partially for the loss of MBNL1 [37]. The same study found that constitutive loss of both MBNL1 and MBNL2 is embryonically lethal. However, they found that Mbnl1^−/−^/Mbnl2^+/−^ were viable (albeit with reduced survival), smaller in size, and had increased myotonia, weakness, and severe myopathy by 8–12 weeks of age. Given the myopathic changes in *Mbnl1*^−/−^*/Mbnl2*^+/−^ mice, and the lack of significant myopathy or changes in Pax7 expression and MuSCs in the individual knockout lines, we decided to evaluate the *Mbnl1*^−/−^*/Mbnl2*^+/−^ mice for these parameters. Because of the weakness and small size of the mice, we could not carry out a BaCl_2_-induced damage experiment in these mice. However, H&E staining of TA muscles from 2-month-old mice showed myopathy in the absence of any BaCl_2_-induced damage, as evidenced by increased central nuclei, small fibers, fiber degeneration, and fiber size variation (Figure 5A,C). Additionally, we found that the number of MuSCs (as assessed by PAX7 IF) and expression of Pax7 mRNA were significantly higher (approximately 2-fold) in the *Mbnl1*^−/−^*/Mbnl2*^+/−^ mice as compared to wildtype mice (Figure 5A,C). The percentage of fibers with central nuclei was approximately 16% (Figure 5C), double that seen in MBNL1-deficient mice (see Figure 2D). Gomori trichrome staining did not show significant fibrotic change in the TA muscles, but there were some patchy areas of increased collagen deposition in the *Mbnl1*^−/−^*/Mbnl2*^+/−^ mice (Figure 5B).

### 2.4. MBNL1 Overexpression and Skeletal Muscle Regeneration

Over a decade ago, the Ranum lab developed mice (MBNL1-OE mice) that overexpress the 40 kDa isoform of human MBNL1, the predominant form of MBNL1 in skeletal muscle [54]. In their study, they showed that these mice could rescue key DM1 related phenotypes (e.g., myotonia and RNA splicing defects) in the skeletal muscles of HSALR mice [54]. In our subsequent study using the DM200 and DM5 mouse models in combination with MBNL1-OE mice, we found differing results [55]. In that study, we also noted that mice with MBNL1 overexpression had increased numbers of MuSCs (quadriceps femoris) and increased Pax7 expression (gastrocnemius/soleus) in their skeletal muscles [55].

**Figure 6:**
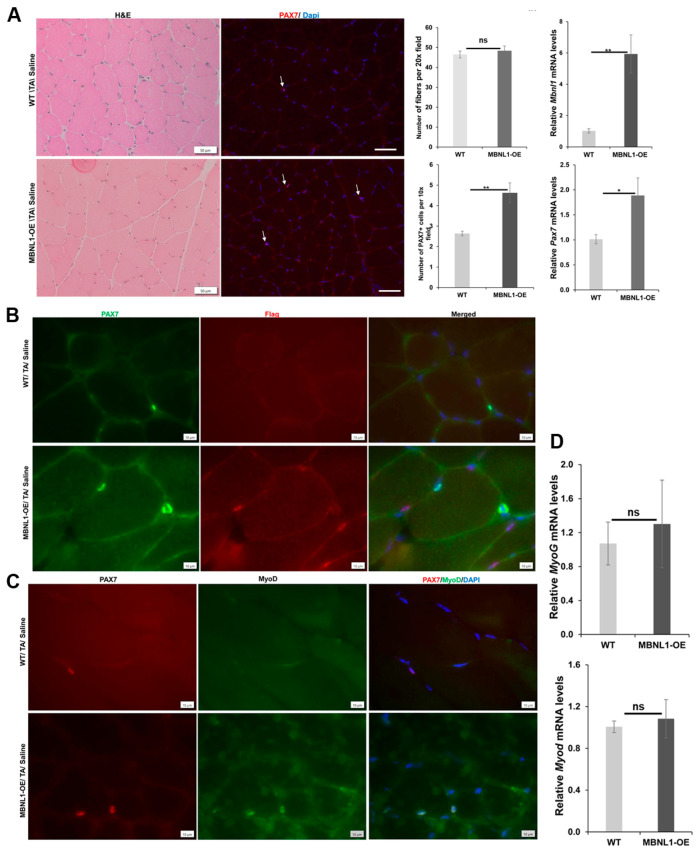
Skeletal muscle is histologically similar between WT (FVB) and MBNL1-OE mice. (**A**) A representative image of H&E staining of TA muscles of non-injured muscle from WT and MBNL1-OE mice, and PAX7 IF (red) in TA muscle, showed increased satellite cells (white arrows) in MBNL1-OE as compared to wildtype mice. RT-PCR of *Mbnl1* mRNA confirmed overexpression in MBNL1-OE mice. Muscle fiber counts showed no difference, and MBNL1-OE mice had higher counts of MuSCs in TA muscles (at least 10 independent fields from *n* ≥ 4 mice/group). RT-PCR showed increased Pax7 expression in MBNL1-OE mice; scale bar is 50 µm. (**B**) Immunofluorescence of recombinant MBNL1 using the anti-Flag rabbit polyclonal antibody (F7425, Sigma) in MBNL1-OE TA muscle showed MBNL1 is not expressed in the satellite cells; PAX7 (green), Flag (red), and nuclei are stained blue. Recombinant MBNL1 protein was not detected in wildtype (WT) controls. Scale bar is 10 µm. (**C**) Immunofluorescence for PAX7 (red) and MyoD (green) in non-injured TA sections from MBNL1-OE mice showed MuSCs co-expressing PAX7 and MyoD (a marker of “activated” MuSCs); this was rarely seen in WT mice; scale bar is 10 µm. (**D**) Quantitative RT-PCR of relative expression, *Myog*, and *Myod* in whole TA muscles (*n* ≥ 4 mice per group) showed no significant difference. Error bars are mean ± SEM; Student’s *t*-test; ns = not significant; * *p* ≤ 0.05, ** *p* ≤ 0.01.

At baseline, prior to induced damage, there was no obvious difference in the histologic appearance or in the number of fibers between WT (FVB) and MBNL-OE mice (Figure 6A). RT-PCR confirmed an overexpression of MBNL1 (Figure 6A). As seen previously, we found a significantly increased number (1.8-fold) of MuSCs in the TA muscles of MBNL1-OE mice (Figure 6A), and RT-PCR confirmed that *Pax7* expression was 1.9-fold higher (Figure 6A). The transgenic MBNL1 protein is expressed with a FLAG tag. However, FLAG-IF in conjunction with PAX7-IF showed that the transgenic protein was not expressed in MuSCs in MBNL1-OE mice (Figure 6B). We assessed the state of the MuSCs by using a combination of PAX7-IF and MyoD-IF since MuSCs in their resting state are PAX7^+ve^/MyoD^−ve^ and activated MuSCs are double positive (Pax7^+ve^/MyoD^+ve^) (Figure 6C). Interestingly, though they were rare, we found more double positive MuSCs in the MBNL1-OE mice (34 of 92 MuSCs in MBNL1-OE vs. 14 of 82 in WT mice) (Figure 6C). This result suggests that the MuSCs in the MBNL1-OE mice were more poised to commit to terminal differentiation into myoblasts. Quantitative RT-PCR of whole TA muscle RNA extracts, however, did not show differences in *Myod* or *Myog* expression (Figure 6C).

**Figure 7:**
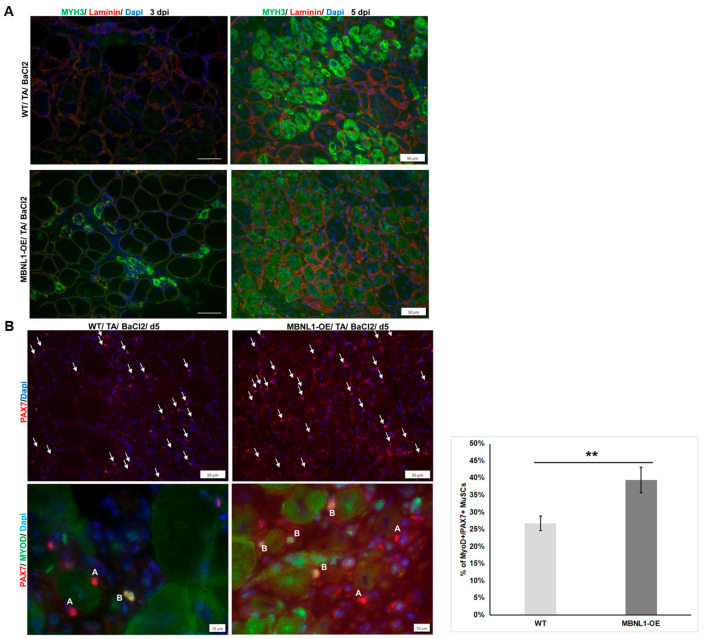
(**A**) Immunofluorescence images show embryonic myosin heavy chain (MYH3) (green) with laminin (red) in regenerating fibers in WT and MBNL1-OE TAs at 3 days (left) and 5 days (right) after BaCl_2_ injury. Nuclei were stained with DAPI (blue). (**B**) Top row: Pax7-IF (red) showed increased numbers of MuSCs (arrows) in WT and MBNL1-OE mice at 5 dpi (top row); scale bar is 50 µm. Bottom row: combined PAX7-IF (red) and MyoD-IF (green) used to detect the status of MuSCs in the TA muscle at 5 dpi; nuclei stained blue with DAPI; scale bar is 50 µm. The letter (A) indicates Pax7^+ve^/MyoD^−ve^ MuSCs and (B) indicates Pax7^+ve^/MyoD^+ve^ MuSCs. Graph depicts the total proportion of MuSCs in activated state (i.e., MyoD+ and Pax7+). Error bar is mean ± SEM.; ** *p* = 0.003 for Student’s *t*-test, *n* = 3–5.

Next, we undertook an evaluation of muscle regeneration in the MBNL1-OE mice using the BaCl_2_-induced damage protocol. Myosin heavy chain 3 (MYH3), an embryonic MYH, is expressed in early stages of regeneration in newly formed myofibers. Its expression typically reaches a peak around 5 days post-injury (dpi) and then subsides as fibers mature and change their MYH isoforms. Immunofluorescence staining for MYH3 revealed numerous fibers expressing MYH3 in WT mice at 5 dpi (Figure 7A), whereas the staining in MBNL1-OE mice showed more fibers expressing MYH3 but at a lesser intensity. This could either represent delayed maturation or more rapid maturation whereby the fibers are towards the end of their MYH3 expression period. Therefore, we collected TA muscles at 3 dpi and found that the MBNL1-OE were already expressing MYH3, whereas this was rarely seen, if at all, in WT mice (Figure 7A). These results are consistent with a more rapid progression in regeneration and consistent with the data that more MuSCs were poised to differentiate (see Figure 6). Similarly, combined PAX7-IF and MyoD-IF showed that at 5 dpi the TA muscles from MBNL1-OE mice had a higher percentage of double positive MuSCs as compared to WT mice (39.4% vs. 26.8%; *t*-test *p* = 0.004) (Figure 7B).

**Figure 8:**
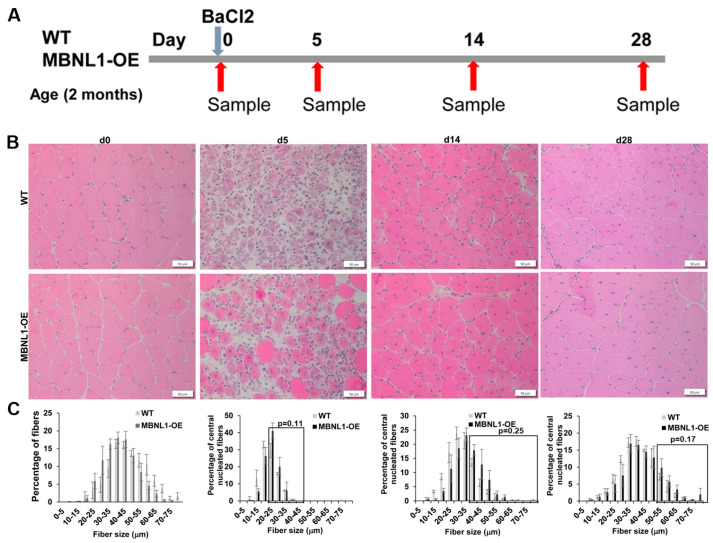
(**A**) Schematic showing the experimental design used in this study. (**B**) Representative H&E staining of TA muscles sections 0, 5, 14, and 28 days post-injury (dpi); scale bar is 50 µm. (**C**) Fiber size distribution analyses in the WT and MBNL1-OE mice; *n* = 3–5 mice per group. At least 3 non-overlapping sections per TA muscle from each mouse were analyzed. Student’s *t*-test used to determine significance. Error bars are mean ± SEM.

**Figure 9:**
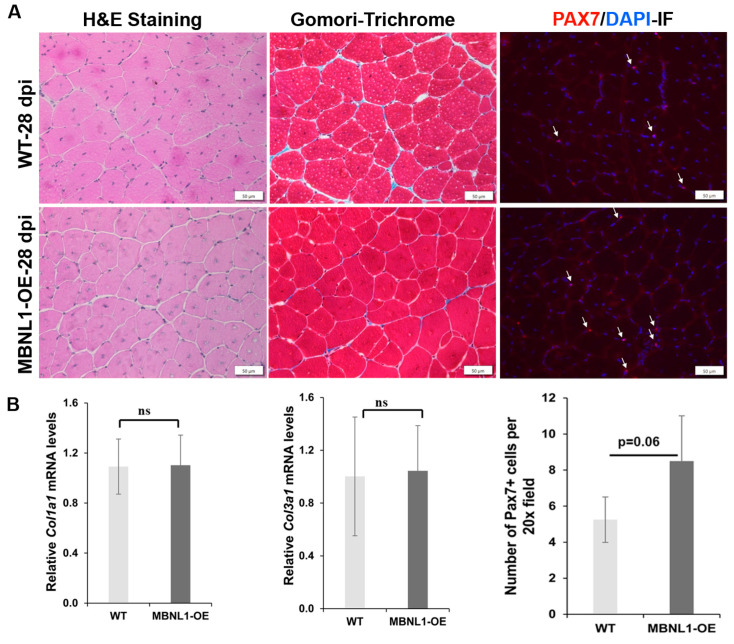
(**A**) H&E, Gomori-trichrome, and PAX7-IF of sections of TA muscles collected after 28 days of regeneration following injury for WT and MBNL1-OE muscles showed similar levels of staining. Scale bar is 50 µm. (**B**) Quantitative RT-PCR showed no significant change in the expression of *Col1a1* and *Col3a1* in MBNL1-OE mice as compared to WT mice, but quantification of MuSCs (PAX7+ve cells) showed elevated levels in MBNL1-OE (*p* = 0.06); *n* ≥ 4 mice per group; error bars are mean ± SEM; scale bars indicated; Student’s *t*-test; ns means not significant.

Fiber size analyses were carried out to compare the regenerative response in MBNL1-OE mice versus WT mice. As mentioned earlier, histologically, the TA muscles were similar in both groups prior to damage (Figure 8). At 5 dpi, fiber sizing and H&E suggested a trend towards increased fiber size in MBNL1-OE mice, but this was not statistically significant. At 14 dpi and 28 dpi, there was no obvious difference in the histologic appearance of the regenerating muscles between the two groups, nor was there a difference in the fiber size distribution. Furthermore, Gomori trichrome staining showed efficient repair with little to no fibrotic change as compared to wildtype mice (Figure 9). RT-PCR for collagen genes (*Col1a1* and *Col3a1*) showed no difference in the expression of these two genes at 28 dpi between the MBNL1-OE and the WT mice (Figure 9). This data suggests that the MBNL1-OE mice repair their damaged TA muscles as well as the WT mice.

## 3. Discussion

The goal of this study was to evaluate muscle regeneration in mouse models of MBNL1 and MBNL2 deficiency in order to assess if their loss in skeletal muscle could account for the regeneration and MuSC defects observed with RNA toxicity [44]. Why is studying muscle regeneration relevant in this context? MBNL1 is the predominant MBNL member in skeletal muscles. Both proteins can co-localize and bind with the (CUG) RNA foci in mouse models as well as in skeletal muscles from individuals with DM1. The prevailing hypothesis invokes sequestration of these proteins by the RNA foci, resulting in functional depletion of MBNLs, and previous work with the mouse models used in this study supports it [36,37]. However, there are no reports of detailed analyses of the consequences of the loss of these proteins on muscle regeneration.

There are several outcomes from the current study. First, two-month-old adult MBNL1-deficient mice show increased muscle histopathology in the absence of any BaCl_2_-induced damage, as evidenced by an increase in central nucleated fibers (>8% vs. <2% in WT mice; Figure 2C) and a distortion towards larger muscle fiber diameters (Figure 2A,B). This is similar to previous reports [36]. Second, MBNL1 deficiency did not affect MuSC numbers nor *Pax7* expression (Figure 2D,E). Third, muscle regeneration in response to BaCl_2_-induced damage was similar between WT and MBNL1 knockout mice. There was equivalent regeneration by 5 days as detected by histology (Figure 3B) and MYH3 IF (Figure 4B). Fiber sizing showed that the MBNL1-deficient mice repaired the muscle, but they repaired it to its baseline pathology (28 days post-damage), with a shift towards larger fibers evident by 14 days after damage (Figure 4B,C). Of note, though we did not quantify it, there seemed to be more fibers with more central nuclei in the MBNL1^−/−^ mice as compared to wildtype mice (see Figure 4B). Fourth, Gomori trichrome staining and RT-PCR for collagen genes showed no evidence of increased fibrosis in the repaired muscles in MBNL1^−/−^ mice and showed no difference in *Pax7* expression (Figure 4). In summary, MBNL1-deficient mice showed a repair response comparable to wildtype mice, with no deleterious effects on the number of MuSCs. Similar studies in MBNL2-deficient mice showed no differences as compared to wildtype mice for all the evaluated parameters. Overall, neither the loss of MBNL1 nor MBNL2 resulted in significant defects in muscle regenerative capacity or satellite cells.

But MBNL2 is upregulated in MBNL1-deficient mice [37] and is thought to compensate for the loss of MBNL1 resulting in minimal myopathic changes. Our analysis confirmed upregulation of MBNL2 protein levels in TA muscles of MBNL1-deficient mice (Figure 1). Of note, we did not see upregulation of *Mbnl2* mRNA levels using RT-PCR primers spanning constitutively expressed exons 2 and 3. This is in contrast to the results published in the original study where they found upregulation of both RNA and protein levels [37]. For the sake of speculative discussion, it could also be due to the generation of circular RNAs that include the targeted exons in the RT-PCR assay [21,22], though how this could happen is unclear. Perhaps it may be related to different muscles being analyzed; TA muscles in our study, and quadriceps muscles in the study by Lee et al. [37]. A recent study showed that alternative splicing resulting in inclusion of exon 6 (54 bp) and exon 9 (95bp) of *Mbnl2* in MBNL1 knockout mice, mediated the upregulation of MBNL2 and its increased nuclear localization through post-transcriptional mechanisms [56]. However, in that study, they too found no transcriptional upregulation of *Mbnl2* mRNA using primers spanning constitutive exons 3 and 4. In this case the study used gastrocnemius muscles. So, it is unresolved if *Mbnl2* transcriptional regulation, in the context of MBNL1 depletion, may depend on muscle type. Based on the study by Nitschke et al. [56], we used qRT-PCR primers in exons 6 and 9 of *Mbnl1* and *Mbnl2* to evaluate if their inclusion changed in the mouse models. Consistent with the results from that study, we found that transcripts with inclusion of *Mbnl2* exon 6 and 9 were about 7.5-fold higher in TA muscles from *Mbnl1*^−/−^ mice (Supplementary Figure S1). The levels of transcripts with inclusion of *Mbnl1* exons 6 and 9 in the *Mbnl2*^−/−^ mice were unchanged as compared to WT mice (Supplementary Figure S1). Regardless, there is consensus on the increase in MBNL2 protein levels in skeletal muscles of MBNL1-deficient mice.

The combined loss of MBNL1 and MBNL2 is embryonically lethal. But *Mbnl1*^−/−^*/Mbnl2*^+/−^ mice are viable, albeit with significant weakness, low weight, and reduced survival [37]. We could not conduct a BaCl_2_-induced muscle damage experiment in these mice, due to their sickness and weakness. However, our analyses of TA muscles from these mice showed a significant amount of muscle histopathology, increased MuSCs in response to the pathology, and patchy but not significant or marked fibrotic change (Figure 5). Thus, even with combined MBNL1 loss and partial MBNL2 depletion, there was upregulation of MuSCs in response to myopathy but limited dystrophic changes. How does this compare to studies on muscles from individuals with DM1 or mouse models of RNA toxicity?

Historically, reports of DM1 muscle histopathology have noted a surprisingly minimal regenerative response despite ongoing dystrophy [39,57]. This is unlike most other forms of muscular dystrophy such as Duchenne muscular dystrophy where evidence of regeneration is present in conjunction with dystrophic changes. In addition, muscle immaturity in congenital DM1 [58,59] and muscle atrophy in adult DM1 have been found in multiple studies.

With respect to effects on MuSCs in DM1, progressive loss of satellite cells has been reported with disease progression in the same patient [60] and in muscles from children with congenital DM1 [58]. Defects in MuSC activation and proliferation have been reported and reviewed as well [61,62,63,64]. Several studies have also reported decreased myogenic potential and differentiation defects in myoblasts and embryonic stem cells from individuals with DM1 [65,66]. CRISPR/Cas9 genome editing of the repeat sequence in the *DMPK* gene of myoblasts derived from individuals with congenital DM1 also resulted in better myogenic differentiation [65]. In addition, several studies have demonstrated increased cellular senescence in DM1 myoblasts that led to decreased proliferative and differentiation capacity [18,20,24].

RNA toxicity and its effects on myogenesis were first demonstrated in our studies using C2C12 mouse myoblasts, where expression of the mutant *DMPK* 3′UTR led to myogenic and myoblast fusion defects that were corrected by either deletion of the repeat sequence or cessation of expression of the mutant transcript [6]. More recently, we showed that RNA toxicity in the DM200 mouse model led to decreased MuSC numbers, impaired regeneration, and decreased response of MuSCs to BaCl_2_-induced skeletal muscle damage [45]. HSALR mice, the prototype mouse model for RNA toxicity, show increased *Pax7* expression and increased MuSCs in young adult mice (2 months old) but decreased *Pax7* expression and MuSC numbers in mice over 6 months of age [13,30]. Similarly, the DMSXL mouse model has also been shown to have decreased *Pax7* expression in adult skeletal muscles [67].

The effects on MuSCs and myogenic differentiation in DM1 and various mouse and cell models of RNA toxicity are clearly different from those seen in mice with MBNL1, MBNL2, or combined MBNL1 loss/MBNL2 deficiency. This is in spite of having a plethora of splicing defects in common (in severity and direction of mis-splicing) with those seen in skeletal muscles of the HSALR mouse and individuals with DM1 and DM2 [68,69]. Notably, unlike myoblasts and satellite cells from individuals with DM1, those from individuals with DM2 showed little evidence of defects in myogenesis [70,71], despite having larger RNA foci and increased MBNL1 sequestration [36,68]. In sum, these studies all point to the notion that factors other than RNA splicing defects and MBNL sequestration play a role in defects in MuSC number and myogenic activation associated with RNA toxicity. This concept has been discussed as early as 2003 by Mankodi et al. [36,68] and reviewed in detail recently [40].

The other question we addressed in the current study was to assess the effects of MBNL1 over-expression on MuSCs and muscle regeneration. This is pertinent, as increasing MBNL1 expression in skeletal muscle has been proposed as a potential therapeutic strategy for RNA toxicity in DM1 [54,68]. We confirmed our previous observation that MBNL1 over-expression in skeletal muscles resulted in increased MuSC numbers [55]. Of note, this seems to be happening in a non-cell autonomous manner since we did not find expression of the transgenic MBNL1 in MuSCs. How this happens is unclear, but our results do suggest that it somehow creates a situation where some of the MuSCs are primed to be in a more “activated” state and committed to differentiation. The result is a regenerative response that progresses faster, that results in repair comparable to wildtype mice by 28 days post-damage, and that restores the higher MuSC numbers by that time compared to wildtype mice. In spite of this, our previous study showed that MBNL1 overexpression led to more severe myopathy in two different mouse models of RNA toxicity due to the expression of the DMPK 3′UTR mRNA (either (CUG)_5_ over-expression or (CUG)_200_) [55].

In conclusion, our study shows that muscle repair in response to induced damage is intact in mice with the loss of MBNL1 or MBNL2. Furthermore, over-expression of MBNL1 does not adversely affect muscle regeneration under these experimental conditions, though it does result in an increase in MuSCs and a more activated state in MuSCs. In conjunction with previous studies, this current study highlights the fact that additional factors, such as various signaling pathways (reviewed in [40]), may play significant roles in muscular dystrophy associated with RNA toxicity.

## 4. Materials and Methods

### 4.1. Experimental Mice

Detailed information on the generation of *Mbnl1^∆E3/∆E3^* and *Mbnl2^∆E2/∆E2^* mice has been published [37]. A WT control (*Mbnl1*^+/+^*, Mbn2*^+/+^, and C57BL/129 background) was used with *Mbnl* knockouts. MBNL1-OE lines (MBNL1-OE14685^+/WT^ and MBNL1-OE14686^+/WT^) were obtained from Dr. Ranum [54]. WT mice (FVB) were used as a control for MBNL1-OE. All animals were used in accordance with protocols approved by the Animal Care and Use Committee at the University of Virginia. Both males and females were used for all experiments.

### 4.2. Muscle Regeneration

For induced muscle damage, at the age of 2 months, 50 μL of 1.2% BaCl2 (Sigma-Aldrich, St. Louis, MO, USA) dissolved in saline was injected into the right tibialis anterior (TA) muscle of mice. The TA muscle in the opposite leg of each mouse was injected with PBS as a control. We injected three or more regions in the TA to try to ensure uniform application of BaCl_2_ or PBS. Mice were euthanized and TA muscles were harvested at various time points as indicated in the results section for protein, RNA analyses, and histological analyses.

### 4.3. H&E and Gomori’s Trichrome Staining of Skeletal Muscles

H&E staining was performed according to standard protocols. Collagen was detected using a Gomori trichrome stain (#87020, Thermo Scientific™ Richard-Allan Scientific™ Gomori Trichrome (Blue Collagen), Waltham, MA, USA). At least three non-overlapping sections per mouse were analyzed, and 3–5 mice per group were analyzed.

### 4.4. Muscle Fiber Analysis

Muscle fiber sizing was performed using CarlZeiss AxioVision^TM^ software (version 4.8.2) by measuring the cross-sectional area of each muscle fiber in digital images of H&E-stained skeletal muscle (tibialis anterior) sections captured at 200× magnification. Counting of fibers was also performed using the same software. For damaged tissues (day 5, 14, and 28 post-injury), we only used fibers with central nuclei for assessing fiber size, as the central nuclei were evidence of a regenerating fiber. At least three non-overlapping sections per mouse were analyzed, and 3–5 mice per group were analyzed.

### 4.5. RNA Isolation and qRT-PCR Assays

We extracted total RNA from skeletal muscle tissues using published protocols [72]. All RNAs were DNAse treated and confirmed to be free of DNA contamination. A QuantiTech^TM^ Reverse Transcription Kit (Qiagen^TM^, Germantown, MD, USA) was used for making cDNA from 1 µg of total RNA. qRT-PCR was conducted using the BioRad iCycler^TM^ (Biorad, Hercules, CA, USA) and detected with SYBER^TM^ Green dye. Data were normalized using an endogenous control (*Gapdh*), and normalized values were subjected to a 2^−ΔΔCt^ formula to calculate the fold changes between groups. Target genes that had a Ct value of more than 35 were considered not detectable and not used for analysis. The primer sequences are described in Supplementary Table S1.

### 4.6. Immunofluorescence and Western Blot

Details about the immunofluorescence protocols are described elsewhere [44]. Primary antibodies were anti-PAX7 (1:50, Developmental Studies Hybridoma Bank, Iowa City, IA, USA), anti-MBNL1 (1;1000, A2764, gift from Dr. Charles A. Thornton), anti-MYH3 (1:200, clone F1.652, Developmental Studies Hybridoma Bank, Iowa City, IA, USA), anti-laminin (1:1000, catalog L9393, Sigma-Aldrich, St. Louis, MO, USA)), anti-MyoD rabbit polyclonal (1:200, SC-760, Santa Cruz Biotechnology Inc., Dallas, TX, USA), anti-Ki67 antibody rabbit polyclonal (#ab15580, Abnova, Taipei, Taiwan), and anti-Flag rabbit polyclonal (F7425, Sigma-Aldrich, St. Louis, MO, USA). Secondary antibodies (1:000 dilution) were from Molecular Probes Eugene, OR, USA. For quantification of PAX7+ cells, at least three non-overlapping sections per mouse were analyzed, and 3–5 mice per group were analyzed.

For Western blot analysis, total protein extracts were made from WT, *Mbnl1*^−/−^, and *Mbnl2*^−/−^ (2 months of age) skeletal muscles (TA) using standard protocols in RIPA lysis buffer (50 mM Tris-HCl, pH 7.4, 150 mM NaCl, 1%NP40, 0.5% Na-deoxycholate, and 0.1% sodium dodecyl sulfate (SDS) and protease inhibitor (cat. #1873580; Roche^®^ Inc., Indianapolis, IN, USA). Proteins were detected with the following antibodies: GAPDH (Ambion #4300, Berlin, Germany), rabbit polyclonal antibody (rpAb) anti-MBNL1 A2764 (gift of C. Thornton, University of Rochester), mouse monoclonal (mAb) anti-MBNL2 3B4 (Santa Cruz Biotechnology, Dallas, TX, USA), and HRP-conjugated anti-mouse, or anti-rabbit, secondary antibody followed by SuperSignal^TM^ West Femto (Thermo Scientific, Waltham, MA, USA).

### 4.7. Statistical Analysis

Standard statistical methods were employed. Briefly, data sets were first analyzed for outliers using Grubb’s test. For real-time PCR, outliers were assessed prior to the calculation of fold change. Once outliers were removed, the data set was analyzed for normality. If normal, the data were analyzed using an unpaired Student’s *t*-test with equal or unequal variance as appropriate. All data are expressed as the mean ± SEM. * *p* < 0.05, ** *p* < 0.01, and *** *p* < 0.001 (Student’s *t*-test). *p* < 0.05 was considered statistically significant unless otherwise specified. ns means not significant.

## Data Availability

Data is contained within the article and Supplementary Material.

## Supplementary Materials

The following supporting information can be downloaded at: https://www.mdpi.com/article/10.3390/ijms25052687/s1.
