# Supplementary material for: Studying the Effect of MBNL1 and MBNL2 Loss in Skeletal Muscle Regeneration
Source: Int J Mol Sci. 2024 Feb 26;25(5):2687. doi: 10.3390/ijms25052687 (PMC10931579; doi:10.3390/ijms25052687)
Supplement: Supplementary file 1 [file ijms-25-02687-s001.zip › ijms-2884215-supplementary.pdf]

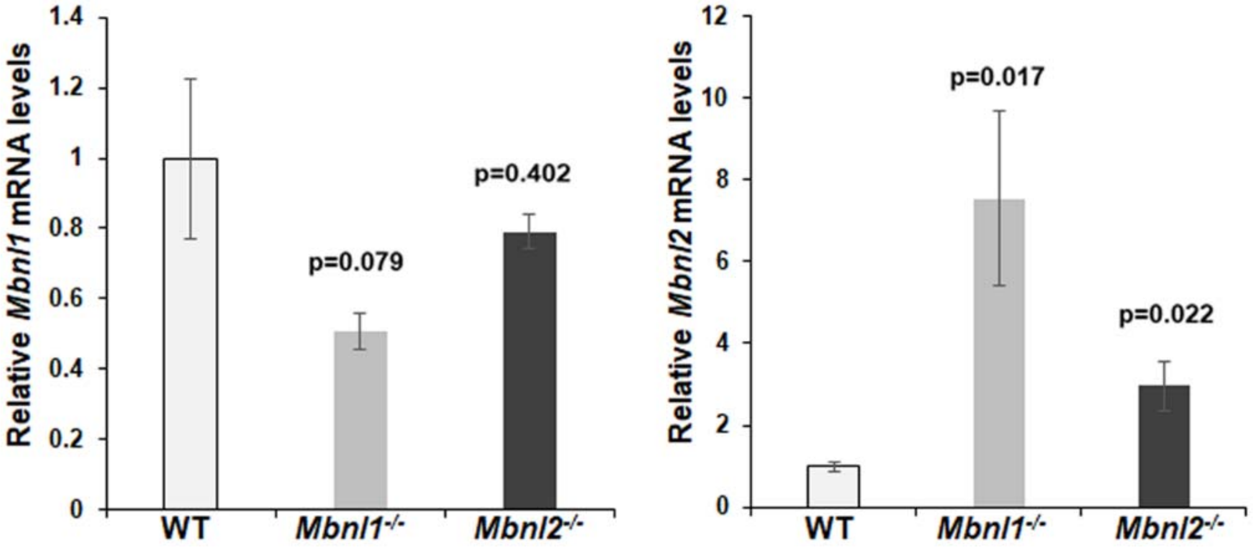

Supplementary Figure S1. Quantitative RT-PCR using primers spanning from exon 6 to exon 9 of *Mbnl1* and *Mbnl2* respectively. RNA from TA muscles. n=4 mice per genotype; error bars are mean ± SEM; p values indicated for Student's T-tests.

Supplementaryl Table S1. Primers for real-time RT-PCR

Gene	Forward primer	Reverse primer	Annealing temp (°C)	PCR efficiency (%)
Gapdh	AGGTCGGTGTGAACGGATTTG	TGTAGACCATGTAGTTGAGGTCA	62	93
Pax7	AGGACGACGAGGAAGGAGACA	TCATCCAGACGGTTCCCTTT	60	98
Myod	ATCCGCTACATCGAAGGTCTG	CTCGACACAGCCGCACTCTTC	62	98
Myog	CCAGTGAATGCAACTCCCACAGC	AGACATATCCTCCACCGTGA	59	102
Col1a1	GAGCGGAGAGTACTGGATCG	GCTTCTTTTCCTTGGGGTTC	61.4	97
Col3a1	GCCCACAGCCTTCTACAC	CCAGGGTCACCATTTCTC	61.4	98
Mbnl1(ex2-3)	CCTTCGAAAAGCTGCCAAGTT	CATGGCCATGTTCTTCTGCTG	62	96
Mbnl2(ex2-3)	CAGGTTGAAAATGGAAGAGTAATT	GCTGCTGCAGTTTTTTGCTG	62	98
Mbnl1(ex6-9)	CATGCAGTTACAGCAGCATAC	GGTATCTGGTTGGCTGTGG	61.4	97
Mbnl2(ex6-9)	CTCGAAGCAACTGTAGACCTG	GCTGTGCATCATGGGTACTG	61.4	96
